# Post‐Intubation Acute Laryngeal Injuries: Analysis of Predictive Factors of Poor Prognosis

**DOI:** 10.1002/lary.32256

**Published:** 2025-05-10

**Authors:** Nicole Elen Lira, Leonardo Palma Kuhl, Paulo José Cauduro Maróstica, Claudia Schweiger

**Affiliations:** ^1^ Programa de Pós‐Graduação em Saúde da Criança e do Adolescente Universidade Federal do Rio Grande do Sul (UFRGS) Porto Alegre RS Brazil; ^2^ Otolaryngology Unit Hospital de Clínicas de Porto Alegre (HCPA) Porto Alegre RS Brazil

**Keywords:** endotracheal intubation, laryngeal stenosis, laryngoscopy, larynx, tracheal stenosis

## Abstract

**Objective:**

The objective of this study was to review the endoscopic management of post‐intubation acute laryngeal injuries in children, the outcome of these treatments, and the possible factors for a poor outcome.

**Methods:**

The study included all pediatric patients aged 0–14 years with an endoscopic diagnosis of post‐intubation acute laryngeal injuries, treated at the Hospital de Clínicas de Porto Alegre from January 2018 to December 2022. The endoscopic techniques used for the treatment of injuries were balloon laryngoplasty and reintubation with a smaller tube with antibiotic and corticosteroid ointment coating around the tube. Possible predictive factors for poor prognosis and the need for a tracheostomy were investigated by reviewing medical records.

**Results:**

Of the 59 patients included, 50 (84.74%) comprised the “success” group and 9 (15.25%) the “failure” group. Predictive factors for poor prognosis are: short time for symptoms returning between airway endoscopic interventions, number of reintubations with antibiotic and corticosteroid ointment, number of airway endoscopies with interventions, and number of total airway endoscopies. Furthermore, when evaluating lesion types found in airway endoscopy, severe glottic edema, subglottic edema, and posterior glottic stenosis were shown to be associated with a worse prognosis.

**Conclusion:**

This is the first review in literature that adds reintubation with corticosteroid and antibiotic ointment coating around the tube to balloon laryngoplasty for the management of post‐intubation acute laryngeal injuries. Almost 85% of our patients were managed with success with such approaches. Injuries such as generalized edema and posterior glottic stenosis were factors associated with a poor prognosis.

## Graphical abstract

This is the first historical review in the literature that adds reintubation with corticosteroid and antibiotics ointment coating around the tube to balloon laryngoplasty for the management of post‐intubation acute laryngeal injuries. Almost 85% of our patients were managed with success with such approaches. Injuries such as generalized edema and posterior glottic stenosis were factors associated with a poor prognosis.

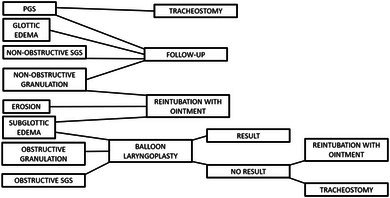

## 1 Introduction

Endotracheal intubation can generate trauma in the glottic and subglottic region and lead to the development of laryngeal injuries of varying degrees. The most frequently encountered acute laryngeal injuries are laryngeal edema, hyperemia, ulcers, granulation tissue, subglottic stenosis (SGS) and vocal fold immobility [1]. Patient conditions can vary from asymptomatic or mild stridor to severe ventilatory difficulties, which can progress to respiratory fatigue and, if untreated, death [2]. The prompt identification of patients with acute laryngeal injuries and their treatment is the best way to prevent chronic injuries and complications.

There have been significant improvements in endoscopic treatment techniques for acute laryngeal injuries in recent years, mainly with the introduction of balloon dilation, which is the main therapeutic tool nowadays [3]. Other endoscopic procedures can also be used as treatment for these injuries, such as removal of granulation tissue and reintubation with corticosteroid and/or antibiotic ointment coating a smaller endotracheal tube. This last therapeutic modality, despite having been described by an *expert* and being used in several airway centers around the world, has not yet been studied, and to date no review with pediatric patients has been found in the literature [4].

The objectives of this study were to describe the management of acute post‐extubation laryngeal injuries at the Hospital de Clínicas de Porto Alegre (HCPA) with balloon laryngoplasty (BLP) and/or reintubation with a tube wrapped in corticosteroid and antibiotics ointment, the outcomes of this approach, and to evaluate the factors related to the failure of the endoscopic treatments.

## 2 Materials and Methods

Symptomatic patients aged 0–14 years with acute post‐extubation laryngeal injuries, included between 2018 and 2022, were selected for this study.

Patients with signs of post‐extubation upper airway obstruction and/or extubation failure due to suspected upper airway obstructive causes underwent airway endoscopy in the HCPA Surgical Center or in the Pediatric Intensive Care Unit (PICU). Laryngoscopies were performed under general anesthesia and spontaneous ventilation with rigid optics (Karl Storz—Germany), with a diameter of 4 mm and zero degrees angle. The devices were connected to the Storz microcamera and the Stryker Vision Elect video monitor and were recorded on DVD. Flexible fiber‐optic laryngoscopy (FFL) was performed for dynamic assessment and diagnosis of associated laryngeal immobility, pharyngomalacia or laryngomalacia.

**FIGURE 1:**
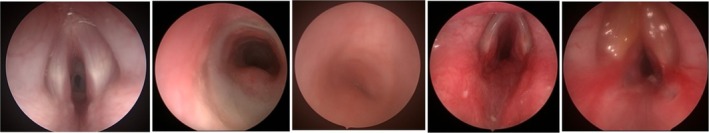
Acute laryngeal injuries. From the left to the right: Granulation; erosion; subglottic edema; posterior glottic stenosis; glottic and subglottic edema.

The endoscopic images obtained were recorded and evaluated by two otorhinolaryngologists with experience in pediatric airways. Findings were classified as follows: erosion; granulation; subglottic edema; glottic edema; posterior glottic stenosis (PGS) and/or laryngeal immobility; and SGS (Figure 1). More than one finding could be found in a patient.

SGS was further graded according to the Myer‐Cotton classification (grades I–IV, grade 1 [< 50% obstruction]; grade 2 [51%–70% obstruction]; grade 3 [71%–99% obstruction]; and grade 4 [complete luminal obstruction]).

**FIGURE 2:**
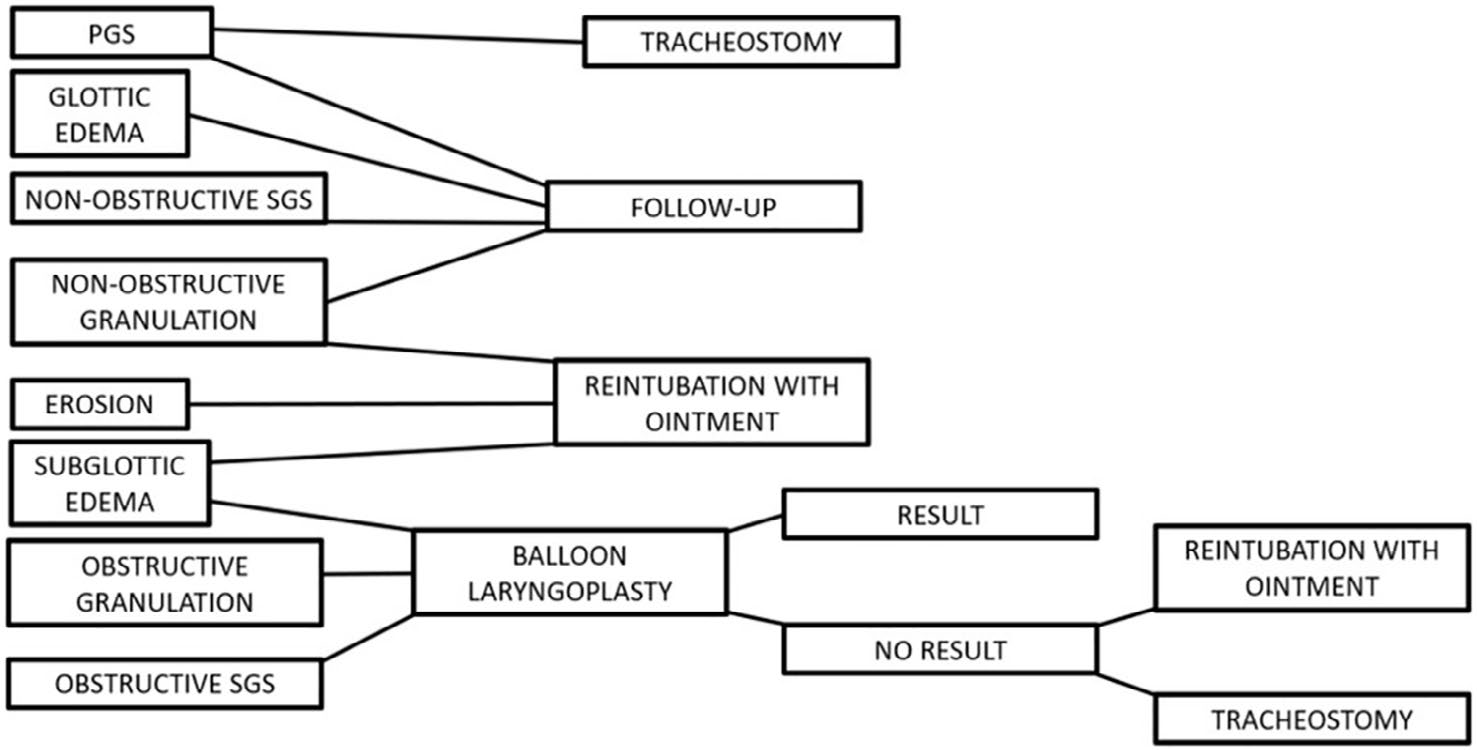
Flowchart of therapeutic management. PGS, posterior glottic stenosis; SGS, subglottic stenosis.

Treatment for each patient was defined according to the lesion found on examination (Figure 2). Observation (close monitoring, without intervention) was chosen when the patient was mildly symptomatic and the following lesions were found in the endoscopic exam: non‐obstructive glottic edema, non‐obstructive SGS, non‐obstructive granulation tissue or mildly symptomatic PGS. When erosion, obstructive subglottic and/or glottic edema, or diffuse non‐obstructive granulation were identified, reintubation was performed with corticosteroid and antibiotic ointment (Betamethasone 0.5 mg + Gentamicin 1 mg—Diprogenta). When there was obstructive granulation tissue and/or obstructive SGS, BLP was performed, and the need for an additional procedure (intubation with ointment) was reassessed according to the immediate laryngoscopic looking in response to treatment. Tracheostomy (TCT) was performed in patients with severely symptomatic PGS, patients who did not respond to the initial instituted treatment, and/or were not clinically stable enough to undergo further attempts. Even patients who required tracheostomy continued to undergo dilations and endoscopic reassessments, in order to determine the need of an open surgery or the possibility of decannulation without further procedures.

When BLP was indicated, the size of the balloon was chosen according to the patient's age and was kept inflated between 30 s and 2 min, or until desaturation occurred; up to two dilations were performed per procedure. When there was an immediate successful response to the procedure, the patient was kept extubated and remained under observation in the PICU for at least 24 h.

**FIGURE 3:**
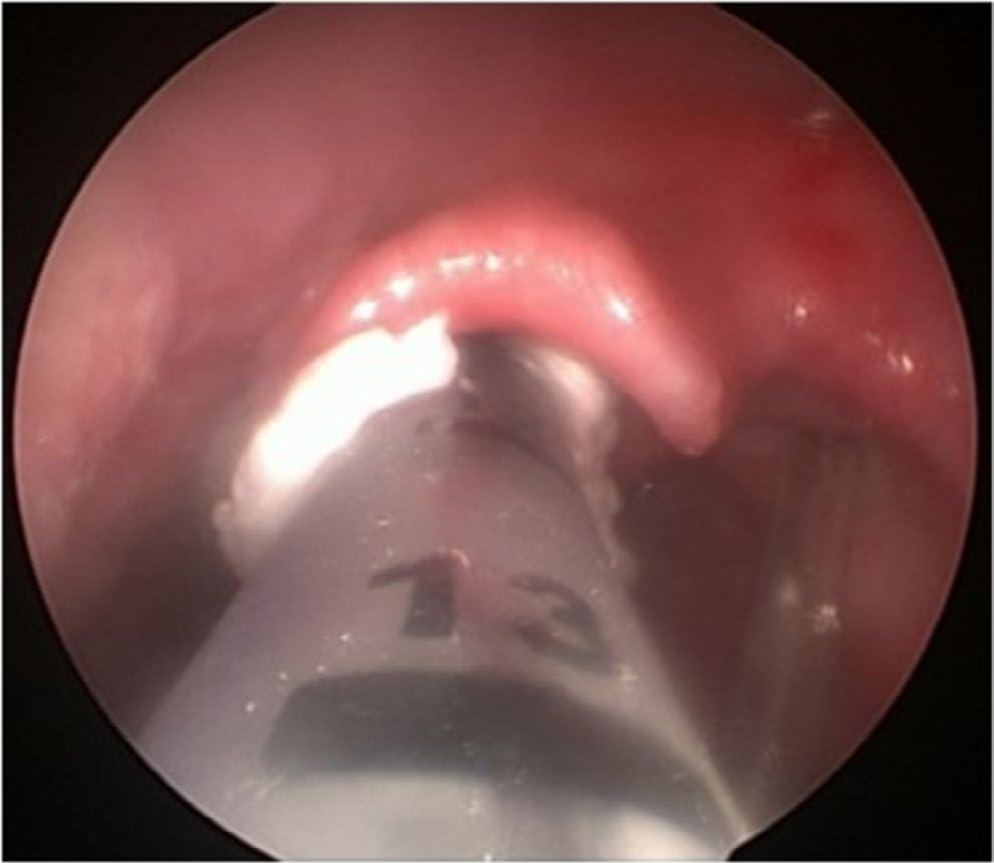
Reintubation with betamethasone + gentamicin ointment.

When reintubation was indicated, patients underwent the procedure with the smallest endotracheal tube indicated for their age, without a cuff and wrapped in Betamethasone + Gentamicin ointment (Figure 3). In our protocol, all patients were reintubated orotracheally. After the procedure, patients were sent to the PICU and maintained with adequate sedation with the aim of decreasing mobilization of the tube, thereby preventing an additional risk factor for acute injury. After around 48 h, according to sedation conditions and clinical readiness for extubation, the patient was extubated.

All patients received a proton pump inhibitor and corticosteroids postoperatively (48 h of intravenous dexamethasone and then 72 h of nebulized dexamethasone). The need for a new laryngoscopy was determined based on the symptoms presented by the patients.

### 2.1 Outcomes and Predictive Factors of Poor Prognosis

“Failure” of the established treatment was defined as the need for tracheostomy.

“Success” was defined when, at the end of treatment, tracheostomy was avoided and the patient evolved without respiratory signs of upper airway obstruction, even with residual stenosis and regardless of the final appearance of the airway.

A comparison was made between the “success” and “failure” groups to evaluate determinants of poor prognosis and consequent treatment failure. The evaluated data were: length of intubation, reason for intubation, appropriate tube size for age, period between extubation and development of symptoms, time for symptoms returning after laryngoscopy, number of total airway endoscopies, number of airway endoscopies with interventions, treatment modality used, and the types of acute laryngeal injuries present at airway endoscopy.

The clinical outcome refers to the patient's situation at the end of the study follow‐up period; all patients had a follow‐up of at least 6 months after the last laryngoscopy.

This study was approved by the research ethics committee of HCPA under the number CAAE 64001722200005327.

### 2.2 Statistical Analysis

Qualitative or categorical variables were described by absolute number and percentage. Continuous variables were described as mean ± standard deviation if normally distributed, or median and interquartile range if asymmetrical distribution. To compare medians, according to success or failure, the Mann–Whitney test was applied. Comparison of proportions was performed using the Chi‐square or Fisher's exact tests. To control for confounding factors, the Poisson regression model was used. The criterion for entering the variable into the multivariate model was that it had a *p* value < 0.10 in the univariate analysis and was considered clinically significant by the researchers. To avoid the effect of multicollinearity, some variables were excluded from the final model (airway endoscopy until final outcome and glottic edema). The significance level adopted was 5% and the analyses were carried out using SPSS version 27.0.

## 3 Results

Fifty‐nine patients were included in the study. Of these, nine (15.25%) patients were considered “failure” requiring TCT; of these, 3 (33.33%) were able to be decannulated during follow‐up, without performing open reconstructive surgery. The other 50 (84.74%) patients evolved without significant obstructive signs and without TCT at the end of treatment. The clinical data of the two groups, as well as the comparison between the factors that could indicate a poor prognosis, are shown in Table 1. There was no statistically significant difference between the two groups regarding these clinical variables.

**TABLE 1:** Group characteristics and univariate analysis of factors predicting poor prognosis.

	Success *n* = 50	Failure *n* = 9	*p*
Age (months)	4 (3–11)	3 (2–18)	0.908
	[1.57–123.03]	[1.77–154.00]	
Male *n* (%)	28 (56%)	7 (77.8%)	0.297
Comorbidity	33 (66%)	7 (77.8%)	0.704
Prematurity	16 (32%)	3 (33.3%)	1
Pneumopathy	7 (14%)	3 (33.3%)	0.17
Heart disease	4 (8%)	2 (22.2%)	0.224
Gastropathy	7 (14%)	1 (11.1%)	1
Neurological disease	11 (22%)	2 (22.2%)	1
Genetic disease	3 (6%)	1 (11.1%)	0.494
Others	13 (26%)	2 (22.2%)	1
No comorbidity	22 (44%)	3 (33.3%)	0.842
1 comorbidity	19 (38%)	4 (44.4%)	
2 comorbidities	3 (6%)	0 (0%)	
3 comorbidities	3 (6%)	1 (11.1%)	
4 comorbidities	3 (6%)	1 (11.1%)	
No. of comorbidities	1 (0–1)	1 (0–2)	0.525
Cause of intubation			
Respiratory	35 (70%)	7 (77.8%)	1
*Bronchiolitis*	30 (85.7%)	6 (85.7%)	1
Neurological	6 (12%)	1 (11.1%)	1
Surgery	7 (14%)	1 (11.1%)	1
Others	2 (4%)	0 (0%)	1
Age‐appropriate tube	36 (80%)	4 (66.7%)	0.598
Cuff	39 (78%)	6 (66.7%)	0.431
Intubation length (days)	7 (4–11.5)	18 (3.5–19)	0.392
	[0–51]	[0–23]	
Extubation failures	2 (1–3)	3 (2–5)	0.112
	[1–4]	[2–5]	
Upper airway obstructive symptoms	33 (66%)	4 (44.4%)	0.272

*Note*: Quantitative variables are described by median, 25th and 75th percentiles (in parentheses) and minimum and maximum (in brackets). Categorical variables are described by number of patients (*n*) and percentage (%).

Twenty‐two (37.3%) patients had extubation failure as an indication for examination under general anesthesia, and 37 (62.7%) had upper airway obstructive symptoms without the need for immediate intubation.

**FIGURE 4:**
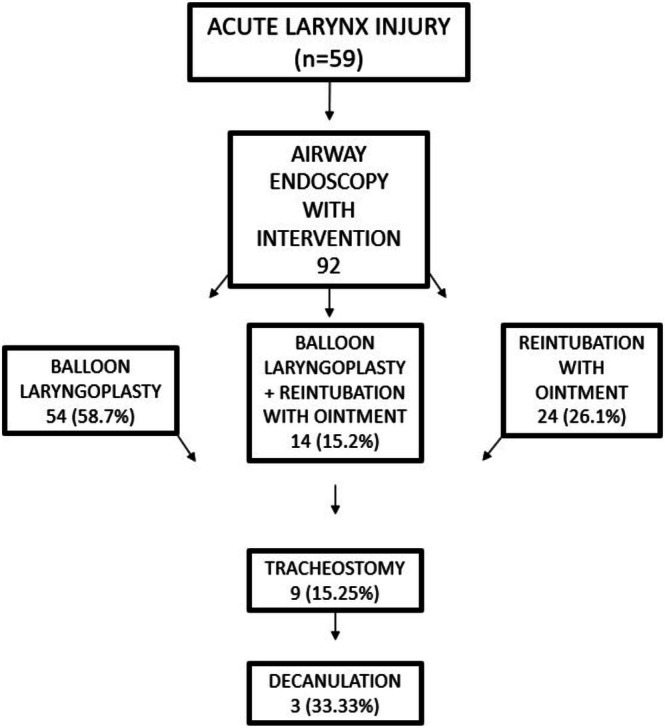
Flowchart of interventions.

At the initial airway endoscopy, 23 (45.1%) patients underwent BLP, 20 (39.2%) patients underwent reintubation with a smaller tube and betamethasone + gentamicin ointment, and 8 (15.68%) patients underwent both procedures. In total, of the 132 laryngoscopies performed, 92 required some intervention: 54 (58.7%) laryngoscopies with balloon dilation, 24 (26.1%) laryngoscopies with reintubation with ointment, and 14 (15.2%) laryngoscopies required both interventions at the same time (Figure 4). Only two patients had complications: both presented laryngospasm and desaturation during airway endoscopy.

Of the 9 patients who underwent tracheostomy, 3 (33.33%) were decannulated without the need for open surgery, 4 (44.44%) were decannulated after open surgery, and 2 (22.22%) remained tracheostomized at the conclusion of this study.

Table 2 shows other clinical factors with a possible poor prognosis.

**TABLE 2:** Airway exams and univariate analysis of predictive factors for poor prognosis.

	Success *n* = 50	Failure *n* = 9	*p*
Number of airway endoscopy until final outcome	2 (1–2)	3 (1.5–4.5)	0.055
	[1–7]	[1–6]	
Number of airway endoscopy with intervention	1 (1–2)	3 (1.5–4.5)	0.003
	[1–6]	[1–6]	
Number of BLP	1 (1–2)	1 (1–4)	0.986
	[1–6]	[1–5]	
Number of reintubation with ointment	1 (1–1)	2 (1–2)	0.014
	[1–2]	[1–3]	
Number of days from extubation until symptoms begin	4 (3–11)	5 (2–18)	0.944
	[2–172]	[2–22]	
Number of days until symptoms return			
Between 1st and 2nd procedure	9 (4–12.5)	1.5 (1–2)	0.007
Between 2nd and 3rd procedure	5 (4.5–28)	1 (1–4)	0.03

*Note*: Quantitative variables are described by median, 25th and 75th percentiles (in parentheses) and minimum and maximum (in brackets). Categorical variables are described by number of patients (*n*) and percentage (%).

Abbreviation: BLP, balloon laryngoplasty.

Reintubation with betamethasone + gentamicin ointment was used in 38 (41.3%) of the airway endoscopy (in 30 (50.84%) patients), with success in 22 patients (73.3%); of these, 14 (63.6%) had reintubation performed alone and 8 (36.4%) with BLP. Decannulation without any open airway surgery was possible in 3 (33.33%) of 9 tracheostomized patients.

When specifically evaluating the lesions found during all the laryngoscopies, we observed that the most common findings were granulation and erosion, presented in 28 (47.4%) and 26 (44%) patients, respectively, followed by glottic edema in 23 (39%) patients, subglottic edema in 17 (28.8%) patients, posterior glottic stenosis in 5 (8.5%) patients, subglottic cyst in 1 (1.7%) patient, and anterior synechiae of the vocal folds in 1 (1.7%) patient.

Subglottic stenosis was classified according to Myer‐Cotton. Thirty‐three (55.9%) patients presented this finding; of these, two (6%) were grade I, five (15.1%) were grade II, and 26 (78.7%) were grade III.

The lesions found during airway endoscopy were evaluated at different times, in order to define whether any specific type of lesion is suggestive of a worse prognosis. For that purpose, we evaluated injury type at the first laryngoscopy and at the last one. When evaluating the first laryngoscopy alone, the presence of glottic edema and subglottic edema proved to be a poor prognostic factor (*p* = 0.008 and *p* = 0.004, respectively). When we evaluated the last laryngoscopy, the presence of glottic edema, subglottic edema, and posterior glottic stenosis showed to be statistically significant (*p* = 0.001, *p* < 0.001 and *p* = 0.01, respectively) (Table 3).

**TABLE 3:** Acute injuries and univariate analysis of endoscopic injuries predictive of poor prognosis.

	Success *n* = 50	Failure *n* = 9	*p*
First laryngoscopy			
Erosion	17 (34%)	6 (66.7%)	0.134
Granulation	18 (36%)	5 (55.6%)	0.292
Glottic edema	6 (12%)	5 (55.6%)	0.008
Subglottic edema	2 (4%)	4 (44.4%)	0.004
Cyst	1 (2%)	0 (0%)	1
PGS	0 (0%)	1 (11.1%)	0.153
SGS	24 (48%)	2 (22.2%)	0.274
Grade 1	1 (2%)	0 (0%)	
Grade 2	4 (8%)	0 (0%)	
Grade 3	19 (38%)	2 (22.2%)	0.505
Last laryngoscopy			
Erosion	14 (28%)	2 (22.2%)	1
Granulation	19 (38%)	2 (22.2%)	0.469
Glottic edema	6 (12%)	6 (66.7%)	0.001
Subglottic edema	2 (4%)	6 (66.7%)	< 0.001
Cyst	1 (2%)	0 (0%)	1
PGS	1 (2%)	3 (33.3%)	0.01
SGS	25 (50%)	4 (44.4%)	1

*Note*: Quantitative variables are described by median, 25th and 75th percentiles (in parentheses). Categorical variables are described by number of patients (*n*) and percentage (%).

Abbreviations: PGS, posterior glottic stenosis; SGS, subglottic stenosis.

Table 4 shows the multivariate analysis of poor prognosis factors. The variables “airway endoscopy with intervention” (*p* < 0.001), “number of reintubations with ointment” (0.016), “presence of subglottic edema” (*p* = 0.031) and “PGS” (*p* < 0.001) showed to be statistically significant. The “number of airway endoscopy until the final outcome” and “glottic edema” were not included in the multivariate assessment, due to the effect of multicollinearity with the variables “number of airway endoscopy with intervention” and “subglottic edema,” respectively. Furthermore, the variable “time between the procedure and the return of symptoms,” which was significant in the univariate analysis, cannot be analyzed in the multivariate due to the fact that the sample size of patients who required more than two procedures was too small. Only four patients required more than three interventions.

**TABLE 4:** Poor prognosis factors and multivariate analysis.

Variables	*N* (95% CI)	*p*
Number of airway endoscopies with intervention	1.83 (0.36–2.47)	< 0.001
Number of reintubation with ointment	2.53 (1.19–5.38)	0.016
Number of subglottic edema	3.59 (1.12–11.5)	0.031
Number of PGS	18.1 (4.26–76.9)	< 0.001

*Note: p* < 0.05 represents significance in multivariate Poisson regression.

Abbreviation: PGS, posterior glottic stenosis.

## 4 Discussion

Endotracheal intubation is one of the most commonly used invasive procedures in the PICU and is associated with different degrees of damage to the laryngeal mucosa [5]. Serious sequelae, such as subglottic stenosis, develop from fundamental injuries such as granulation, edema, ulcerations, and cartilage exposure. The management of acute laryngeal injuries through BLP has already been shown to be effective and, in most cases, capable of preventing the progression to subglottic stenosis, which requires open surgery for its treatment [6]. This is, however, the first study to also describe intubation with a smaller tube and corticosteroid and antibiotic ointment around the tube as an effective treatment for some types of acute laryngeal injury. Management of acute injuries, as described in our protocol, was able to avoid TCT in approximately 85% of cases.

There are few studies that address elective reintubation as a therapeutic option for acute laryngeal injuries, although this technique has been described by *experts* who advocate reintubation with a smaller size ETT, associated with the application of corticosteroid and/or antibiotic ointment coating the tube [7]. This therapeutic modality was used mainly when airway endoscopy showed airway edema and extensive mucosal ulcerations. It was also used when the patient could not tolerate being kept extubated at the end of the balloon laryngoplasty procedure. Furthermore, when patients did not respond immediately to BLP, reintubation with ointment was used as a last attempt before TCT.

Our results are similar to previous studies, such as that by Graham and collaborators, who in 1994 treated 10 newborns with acute laryngeal injury with elective reintubation, associated with adequate sedation, allowing successful extubation in 6 cases [8]. Graham et al., however, only included neonates and did not use ointment around the tube.

In 1989, Bishop suggested that ETT mobilization and pressure on the airway mucosa causing abrasion and necrosis may be risk factors for the development of laryngeal lesions [9]. Also, Monnier states that the child should be kept well sedated during intubation in order to minimize additional laryngeal injuries [7]. Therefore, strategies to avoid or minimize ETT mobilization have been adopted in our study on patients undergoing reintubation with corticosteroid and antibiotic ointment.

In our study, only two patients had complications during laryngoscopy: both presented laryngospasm and desaturations. In both cases, the endoscopy was performed in a PICU bed and not in the Operating Room, and the complications were probably due to the absence of adequate anesthesia, probably indicating that exams carried out in the operating room could bring greater safety and avoid complications.

In the present study, it was not possible to find an association between previous comorbidities, cause of intubation, intubation time, tube size, presence of cuff, and number of extubation failures with a worse outcome of endoscopic treatment of acute laryngeal injuries.

Intubation length is traditionally considered one of the most relevant factors for the development of post‐extubation laryngeal injuries. Manica and collaborators, in 2013, demonstrated that for every five additional days of intubation, there is an average increase in the baseline risk of developing SGS of around 50% [10]. This intubation length, however, does not seem to be associated with the development of more serious acute injuries, which would be more likely to fail endoscopic management.

ETT without a cuff is generally recommended for children under 8 years of age, when the subglottic region is supposed to act as a “functional cuff.” Most of our patients used cuffed ETT; however, we did not find the presence of cuffs to be a significant risk factor for the development of severe laryngeal injuries, in agreement with previous studies [9].

The number of patient comorbidities was also not statistically significant for the development of lesions with a worse prognosis in our study. This prognostic factor had never been studied in acute injuries, but Monnier and collaborators argue that patients with comorbidities associated with fibrous laryngeal stenosis have a worse outcome in reconstruction surgeries [7]. These authors even created a new classification, based on that of Myer and Cotton, where they included comorbidities as a poor prognostic factor.

As expected, the number of procedures with intervention was significantly higher in the “failed” group. Other factors such as time between extubation and onset of symptoms were not statistically significant. The interval between airway endoscopy and the return of obstructive symptoms was significantly longer in the “successful” group (9 vs. 1.5 days after the first laryngoscopy, *p* = 0.007 and 5 vs. 1 day in the second laryngoscopy, *p* = 0 0.03), showing that children with a worse prognosis tend to develop obstructive symptoms again in a shorter period of time compared to those with a favorable prognosis. Therefore, children who experience a return of symptoms within a few days after the first procedure are those most likely to eventually require a TCT. These variables were not included in the multivariate analysis because the number of patients who required more than two procedures was very low, making a reliable statistical analysis of these factors unfeasible.

Finally, the presence of injuries such as subglottic edema, glottic edema, and posterior glottic stenosis was statistically significant for a worse prognosis compared to other acute laryngeal injuries in the univariate analysis. We believe that glottic edema appeared due to the multicollinearity effect, which was confirmed by multivariate analysis. When present by itself on examination, glottic edema does not represent a worse outcome, but it is often associated with subglottic edema, which was shown in this study to be a prognostic factor for an unfavorable outcome.

PGS proved to be statistically significant as a predictor of worse prognosis in multivariate analysis. In the univariate analysis, it was only significant in the last exam; probably, this lesion was not statistically significant at the first laryngoscopy due to the fact that it is a later finding during the development of acute lesions—it may take days to weeks after extubation to develop and become clinically relevant (it gradually fixes the folds vocals, as fibrosis develops).

Corticosteroid injections were not part of our protocol for the treatment of acute lesions; we reserved their application for mature stenoses with a fibrotic appearance. This could probably be another therapeutic tool for acute lesions as well, as already used in some airway centers.

In our protocol, all patients were reintubated orotracheally. We use nasotracheal intubation after reconstructive surgeries only, since we feel that this could help keeping the posterior graft on place and help decreasing the need for sedation along the prolonged postoperative period of intubation that these children warrant. For acute lesions, we do not feel this would change the outcomes (there are no grafts and no prolonged length of intubation). But this is another issue that could well justify a randomized clinical trial, in order to clarify the role of nasotracheal intubation.

## 5 Conclusion

This is the first study to describe a historical review of children undergoing management of acute post‐extubation laryngeal injuries through balloon laryngoplasty and elective reintubation with a smaller ETT, wrapped in betamethasone + gentamicin ointment, followed by a short period of sedation.

Furthermore, it is the first study that evaluates the prognostic factors that may be associated with a worse outcome from these treatments. We observed in this study that some injuries can be predictors of a worse prognosis when evidenced on airway examination, such as generalized edema and posterior glottic stenosis. Furthermore, patients who require procedures with a shorter time between them also have a greater chance of failing endoscopic treatment.

In this initial study, our objective was to evaluate our acute laryngeal injury protocol and to show the outcomes. Further studies are necessary to clarify the risk factors for a poor prognosis, and also to evaluate the role of the ointment‐wrapped endotracheal tube versus intubation only. This latter question warrants a randomized clinical trial.

## Conflicts of Interest

The authors declare no conflicts of interest.
